# Multilocus sequence typing of *Giardia duodenalis* genotypes circulating in humans in a major metropolitan area

**DOI:** 10.3389/fmed.2022.976956

**Published:** 2022-10-05

**Authors:** Saeideh Hashemi-Hafshejani, Ahmad Reza Meamar, Maryam Moradi, Nasrin Hemmati, Shahram Solaymani-Mohammadi, Elham Razmjou

**Affiliations:** ^1^Department of Parasitology and Mycology, School of Medicine, Iran University of Medical Sciences, Tehran, Iran; ^2^Laboratory of Mucosal Immunology, Department of Biomedical Sciences, School of Medicine and Health Sciences, University of North Dakota, Grand Forks, ND, United States

**Keywords:** *Giardia duodenalis*, multilocus sequence typing, triosephosphate isomerase *(tpi)*, β-giardin *(bg)*, glutamate dehydrogenase *(gdh)*, Tehran, Iran

## Abstract

*Giardia duodenalis* is an intestinal protozoan parasite of humans and animal hosts and comprises eight microscopically indistinguishable molecularly-diverse lineages designated as assemblages A–H. Assemblages A and B are the primary sources of infections in humans and a wide range of mammals. Here, we identified assemblages, and inter-/intra-assemblage genetic diversity of human *G. duodenalis* isolates based on the multilocus sequence typing of the triosephosphate isomerase (*tpi*), β -giardin (*bg*), and glutamate dehydrogenase (*gdh*) loci. Multilocus sequence analysis of 62 microscopically-positive *G. duodenalis* fecal samples identified 26 (41.9%), 27 (43.5%), and nine (14.5%) isolates belonging to assemblages A, B, and discordant assemblages, respectively. The *tpi* locus assemblage-specific primers identified dual infections with A and B assemblages (45.2%). The sequence analysis of multiple alignments and phylogenetic analysis showed low genetic polymorphism in assemblage A isolates, classified as sub-assemblage AII at three loci, subtype A2 at *tpi* and *gdh* loci, and subtype A2 or A3 at *bg* locus. High genetic variations were found in assemblage B isolates with 14, 15, and 23 nucleotide patterns at *tpi, bg*, and *gdh* loci, respectively. Further concatenated sequence analysis revealed four multilocus genotypes (MLG) in 24 assemblages A isolates, two previously-identified (AII-1 and AII-5), with one novel multilocus genotype. However, the high genetic variations observed in assemblage B isolates among and within the three genetic loci prevented the definitive designation of specific MLGs for these isolates. Multilocus sequence typing may provide new insight into the genetic diversity of *G. duodenalis* isolates in Tehran, suggesting that humans are likely a potential source of *G. duodenalis* infection. Further host-specific experimental transmission studies are warranted to elucidate the modes of transmission within multiple host populations.

## Introduction

*Giardia duodenalis* (syn. *Giardia lamblia, Giardia intestinalis*) is a flagellate protozoan parasite that infects the small intestine of a wide range of mammals, including humans (1, 2). *Giardia duodenalis* is one of the most prevalent enteric protozoan parasites globally, with prevalence rates varying from 0.4 to 7.5% in developed countries to 8–30% in the developing world (3, 4). The *G. duodenalis* infections are initiated by ingesting quadrinucleate infective cysts through food or water contaminated with feces from infected humans or animals (3). Asymptomatic *G. duodenalis* infections are common in humans and, in most cases, are self-limited and cleared within weeks of exposure with no treatments (4, 5). Asymptomatic infections can lead to a malabsorption syndrome, characterized by failure to thrive and/or stunted growth, especially in children in developing countries. However, infected individuals with the symptomatic disease typically exhibit gastrointestinal symptoms, including diarrhea, abdominal discomfort, flatulence, nausea, and bloating (1, 4–6).

The *G. duodenalis* complex comprises eight morphologically-indistinguishable genotypes with molecularly diverse lineages designated as assemblages A–H (7, 8). *Giardia duodenalis* assemblages A and B infect humans and a variety of other mammals. Whereas, assemblages C–H are commonly found in dogs and other canids (C, D), hoofed livestock (E), cats (F), rodents (G), and marine mammals (H) (3, 4, 7). However, recent accumulating evidence has demonstrated that those assemblages thought to be only circulating within the livestock (i.e., assemblage E) could also infect humans (9–11). This suggests that host-specificity for at least some assemblages is less strict, and those adapted to non-human mammals might be able to infect humans. An allozyme analysis designated four sub-assemblages within assemblages A and B (AI–AIV and BI–BIV), of which AI, AII, BIII, and BIV have been mainly identified in humans (7, 12). Subsequent nucleotide sequence and phylogenetic analysis have confirmed sub-assemblages AI–AIII within assemblages A, with AI being isolated mainly from animals, whereas AII is predominantly identified in humans. Moreover, AIII is mostly reported in wild mammals (e.g., deer), with only two human cases, which have been recently reported (2, 4, 11, 13). In addition, multilocus sequence typing (MLST) has characterized 9–12 subtypes/genotypes at each of the individual loci within the three major sub-assemblages A (4). However, the phylogenetic analysis of the nucleotide sequences of the main used genetic loci has not identified distinct sub-assemblages within assemblage B, likely reflecting a high sequence diversity within this group not supported by the bootstrap analysis (2, 14). Assemblages A and B of *G. duodenalis* possess a wide range of mammalian hosts, including humans. Thus, infections with these two assemblages are potentially considered of zoonotic importance (2, 4).

Multiple genetic markers have been routinely employed to discriminate better the genetic diversity and the population dynamics within a given *G. duodenalis* assemblage (3, 7). To this end, the small subunit ribosomal RNA (*SSU*-rRNA), glutamate dehydrogenase (*gdh*), triosephosphate isomerase (*tpi*), and β-giardin (*bg*) genes are among the most commonly-used loci to identify multiple variants of *G. duodenalis* in different host species (3, 7). The *SSU*-rRNA gene is a multi-copy and highly-conserved locus, making it a feasible genetic surrogate for detecting and differentiating *G. duodenalis* assemblages. In contrast, this locus is considered less useful in identifying intra-assemblage genetic diversity due to its conserved nature and usually short amplified fragments in most PCR assays based on the *SSU*-rRNA locus (2). In contrast to the *SSU*-rRNA locus, the single-copy *tpi, bg*, and *gdh* loci are more sensitive to probing the genetic variation and the classification of *G. duodenalis* populations at the sub-assemblage and genotype levels. However, these loci are not considered feasible candidates for diagnosing *G. duodenalis* in clinical settings (8, 15). Despite existing consensus over the feasibility of these loci to genetically classify multiple *G. duodenalis* assemblages, conflicting findings have been reported regarding the usefulness of a given single locus in differentiating *G. duodenalis* populations into assemblages and sub-assemblages (3, 16). Therefore, to expand accuracy, a numeric multilocus genotyping (MLG) system was introduced using *tpi, bg*, and *gdh* genes in analysis simultaneously (17).

*Giardia* is still considered the most-identified intestinal parasite in Iran, although its overall prevalence rates have dramatically decreased in recent years (18–20). The molecular characterization of the human *G. duodenalis* isolates in Iran was predominantly conducted using a single locus (21–26). In the current study, however, we employed an MLST approach to fill a gap in our understanding of the population structure, and genetic diversity of *G. duodenlais* isolates circulating in a major metropolitan area in Iran.

## Materials and methods

### Study subjects and DNA preparation

From June to November 2015, 41 fecal samples positive for *G. duodenalis* cysts by microscopy were collected from individuals referred to health centers in Tehran for routine stool screenings. Furthermore, archival DNA specimens from an additional 21 fecal samples positive for *G. duodenalis* cysts (2009–2014) were also included for further analyses.

In total, 62 *G. duodenalis* isolates from infected individuals were included in the current study, of which 42 (67.74%) and 18 (29.03%) were males and females, respectively. However, the genders of two participants (3.23%) were not determined. The mean age was 37.1 ± 20.9 years, ranging from 3 to 86 years. Gastrointestinal symptoms were reported by 12 (19.4%) of 62 participants, while 50 (80.6%) were asymptomatic. The most common gastrointestinal symptoms, including diarrhea (*n* = 10), cramps (*n* = 9), abdominal pain (*n* = 8), nausea (*n* = 5), vomiting (*n* = 3), flatulence (*n* = 6), anorexia (*n* = 6), and constipation (*n* = 4), were reported. Collected stool samples were immediately transferred to the research laboratory of the Department of Parasitology and Mycology, School of Medicine, Iran University of Medical Sciences at 4°C for further laboratory examination on the same day.

The presence of *G. duodenalis* cysts in fresh fecal samples was confirmed by light microscopy or a formalin-ether concentration method on a pea-sized piece of fecal samples, followed by further *G. duodenalis* cysts purification using a sucrose flotation gradient technique on the remaining fresh samples (27) to achieve adequate quantity and quality of *G. duodenalis* DNA for sequencing (5, 28). Briefly, 10 g of fresh feces was added to 50 mL of PBS (pH 7.4) and thoroughly mixed. The fecal suspension was passed through three layers of clean gauze, followed by centrifugation at 800 × *g* for 5 min. The sediments were re-suspended in 50 mL of PBS, and 25 mL of the suspension was layered over 20 mL of 1M sucrose solution (specific gravity = 1.13) in a clean 50-mL conical tube and were further centrifuged at 800 × *g* for 5 min. The interface and the upper layer were carefully transferred to a clean 50-mL conical tube and centrifuged at 800 × *g* for 5 min. The fecal pellets were washed three times with PBS and re-suspended in 0.4 mL of PBS containing 2% polyvinylpolypyrrolidone (PVPP). The Purified cysts were kept at −80°C for 24 h before DNA extraction. The DNA was extracted from purified cysts using a QIAamp DNA Mini Kit (QIAGEN, Germany) according to the manufacturer's instructions with some modifications (29). The extracted DNA was stored at −20°C for further analysis.

### Multilocus genotyping of *G. duodenalis* isolates

#### Nested PCR amplification of the *tpi* and *bg* Loci

A 530-bp fragment of the *tpi* locus was specifically amplified using external forward and reverse primers AL3543 and AL3546 and internal forward and reverse primers AL3544 and AL3545, respectively (Table 1) (30). Both primary and secondary PCR reactions were performed in 50 μL volume, containing 25 μL of 2 × Taq DNA Polymerase Master Mix RED (Amplicon III, Copenhagen, Denmark), 0.2 μM of each primer, and 2 μL of template DNA. The amplification scheme consisted of an initial denaturation step at 95°C for 5 min, 35 amplification cycles at 94°C for 45 s, 50°C for 45 s, 72°C for 60 s, with a final extension at 72°C for 10 min. In the second PCR, the annealing temperature was increased to 58°C, whereas other parameters were left unchanged (36).

**Table 1:** Primer sequences and target genes used for molecular identification of *Giardia duodenalis* assemblages and multilocus sequence genotyping.

**Target gene**	**Nested PCR primer designation and nucleotide sequences (5** ^**′**^ **-3** ^**′**^ **)**		**Amplicon size (bp)**	**References**
	**External primers**	**Internal primers**		
*tpi*	*AL3543: AAATIATGCCTGCTCGTCG	AL3544: CCCTTCATCGGIGGTAACTT	530	(30)
	AL3546: CAAACCTTITCCGCAAACC	AL3545: GTGGCCACCACICCCGTGCC		
tpi**		Af: CGCCGTACACCTGTCA	332	(31, 32)
	AL3543: AAATIATGCCTGCTCGTCG	Ar: AGCAATGACAACCTCCTTCC		
	AL3546: CAAACCTTITCCGCAAACC	Bf: GTTGTTGTTGCTCCCTCCTTT	400	
		Br: CCGGCTCATAGGCAATTACA		
*gdh*	GDHeF: TCAACGTYAAYCGYGGYTTCCGT	GDHiF: CAGTACAACTCYGCTCTCGG	432	(33)
	GDHiR: GTTRTCCTTGCACATCTCC	GDHiR: GTTRTCCTTGCACATCTCC		
*β-giardin*	G7: AAGCCCGACGACCTCACCCGCAGTGC	BG511F: GAACGAACGAGATCGAGGTCCG	511	(34, 35)
	G759: GAGGCCGCCCTGGATCTTCGAGACGAC	BG511R: CTCGACGAGCTTCGTGTT		

*I, Inosine binds to all the four bases; **tpi A and B assemblage primers, primers Af and Ar amplify assemblage A and Bf and Br amplify assemblage B.

Mixed infections with assemblages A and B (A+B) were identified by amplifying the *Giardia tpi* gene using a nested-PCR protocol described elsewhere (31, 32). The primary PCR reaction was performed as described above, whereas the second PCR reaction was conducted using primers Af and Ar for assemblage A and Bf and Br for assemblage B (Table 1). These primers are designed to amplify a 332-bp and a 400-bp fragment within the *tpi* locus of assemblages A and B, respectively. The secondary PCR was accomplished in 50 μL volumes with 25 μL of 2 × Taq DNA Polymerase Master Mix RED (Amplicon III, Copenhagen, Denmark), 1–2 μL of the first PCR product as template DNA, and 0.2 μM of each primer (assemblage A) or 0.4 μM (assemblage B). The amplification strategy consisted of an initial denaturation step at 95°C for 5 min, 35 cycles at 94°C for 45 s, 64°C (assemblage A) or 62°C (assemblage B) for 45 s, 72°C for 60 s, followed by a final extension at 72° C for 10 min (36).

A 511-bp fragment within the *bg* gene was amplified using external and internal forward and reverse primers G7, G759, BG511F, and BG511R (Table 1) (34, 35). The primary and secondary PCR reactions were performed in 50 μL volume, containing 25 μL of 2 × Taq DNA Polymerase Master Mix RED (Amplicon III, Copenhagen, Denmark), 0.2 μM of each primer, and 2 μL of template DNA. The amplification scheme consisted of an initial denaturation step at 95°C for 5 min, 35 cycles at 95°C for 30 s, 65°C for 30 s (55°C for secondary PCR), 72°C for 30 s, and a final extension at 72°C for 7 min (36).

#### Semi-nested PCR amplification of the *gdh* locus

A 432-bp fragment of the *gdh* gene was amplified using external forward and reverse primers GDHeF and GDHiR and internal forward primer GDHiF and reverse primer GDHiR (Table 1) (33). The primary and secondary PCR reactions were performed in 50 μL volume, containing 25 μL of 2 × Taq DNA Polymerase Master Mix RED (Amplicon III, Copenhagen, Denmark), 0.5 μM of each primer, and 2 μL of template DNA. The amplification scheme consisted of an initial step at 94°C for 3 min, 1 cycle at 94°C for 2 min, 61°C for 1 min, and 68°C for 2 min, followed by 30 amplification cycles at 94°C for 30 s, 61°C for 20 s, 68°C for 20 s and a final extension at 68°C for 7 min. The secondary PCR amplification consisted of an initial step at 94°C for 3 min, 1 cycle at 94°C for 2 min, 60°C for 1 min, and 65°C for 2 min, followed by 15 amplification cycles at 94°C for 30 s, 60°C for 20 s, 65°C for 20 s with a final extension at 65° C for 7 min.

All PCR reactions were performed using a Gene Atlas thermocycler (Astec Co., Ltd, Fukuoka, Japan). The DNA obtained from a *Giardia* reference strain (ATCC^®^ Number, 30888™) and sterile distilled nuclease-free water were included as positive and negative controls, respectively. The PCR products were fractionated on a 1.5% (W/V) agarose gel (SinaClon, Tehran, Iran) in Tris-acetate-EDTA (TAE) buffer, stained with ethidium bromide (0.05 mg/mL), and were visualized under UV illumination (GeneFlash, Syngene Bio-Imaging, Cambridge, UK).

### Sequence and phylogenetic analysis

The nested- or semi-nested PCR products for each locus were excised and gel-purified using a MinElute Gel Extraction Kit (Qiagen, Hilden, Germany) and were subjected to sequence analysis in both directions (Macrogen, Korea). The DNA sequences were viewed and read by the CHROMAS software (Technelysium Pty Ltd., Queensland, Australia) and further aligned and assembled with the DNASIS MAX program (v. 3.0; Hitachi, Yokohama, Japan). The DNA sequences were blasted (http://blast.ncbi.nlm.nih.gov) to compare homology against DNA sequences deposited in GenBank. The DNA sequences from the *tpi, bg*, and *gdh* loci were combined to achieve concatenated sequence for each *G. duodenalis* isolate successfully amplified at the three loci (37).

The phylogenetic analysis was performed in MEGA X (www.megasoftware.net) using the maximum likelihood (ML) with evolutionary distances calculated by the best-fitting model to describe a robust estimate of the evolutionary distances. Models with the lowest Bayesian Information Criterion (BIC) scores best describe the substitution pattern. In addition, bootstrap analysis was performed with 1,000 replicates to evaluate the reliability of clusters. The sequences obtained from this study were deposited in the GenBank under the accession numbers LC183913–LC183966, LC184067–LC184028, and LC184423–LC184474 for *tpi, bg*, and *gdh*, respectively.

### Statistical analysis

The demographics and the association between symptomatic and a given *G. duodenalis* assemblages were analyzed using SPSS 24.0 software (SPSS Inc., Chicago, IL, USA), and data were presented with 95% confidence intervals.

## Results

### *Giardia duodenalis* assemblage identification

The multilocus sequence analysis of 62 *G. duodenalis*-positive fecal samples using the *tpi, bg*, and *gdh* genes identified 26 isolates as assemblage A (41.9%), and 27 isolates (43.5%) as assemblage B, whereas nine *G. duodenalis* isolates (14.5%) showed inconsistent assemblage classification, also referred to as “discordant assemblages” (Table 2). Using primers targeting the *G. duodenalis tpi* locus of both assemblages A and B, we found 9 (14.5%) and 23 (37.1%) isolates as assemblages A and B, respectively. However, 28 (45.2%) clinical samples harbored both assemblages A and B (Tables 2, 3).

**Table 2:** Assemblages (subassemblage-subtype) A and B identification based on *tpi, bg*, and *gdh* loci and mixed A and B infections according to *tpi* A and B assemblage-specific primers.

**Isolate**	***tpi***	***bg***	***gdh***	***tpi*-mixed**	**Isolate**	***tpi***	***Bg***	***gdh***	***Tpi*-mixed**
IGT1	B (BIII/BIII-like)	B (BIV-B3)	B	B	IGT32	B (BIII)	B (BIV)	B	A+B
IGT2	A (AII-A2)	A (AII-A3)	A (AII-A2)	A+B	IGT33	B (BIII-like)	B (BIV)	–	B
**IGT3**	**A (AII-A2)**	**B (BIV-B3)**	**–**	B	IGT34	B (BIII/BIII-like)	B (BIV)	B	B
**IGT4**	**B**	**B (BIV-B6)**	**A (AII-A2)**	B	IGT35	B (BIII)	B (BIV-B3)	B	A+B
**IGT5**	**A (AII-A2)**	**B (BIV)**	**–**	B	IGT36	B (BIII)	B (BIV)	B	B
IGT6	–	B (BIV-B3)	–	B	IGT37	A (AII-A2)	A (AII-A3)	A (AII-A2)	A
IGT7	B (BIII)	B (BIV)	B	A+B	**IGT38**	**–**	**B (BIV)**	**A (AII-A2)**	**–**
IGT8	A (AII-A2)	A (AII-A2)	A (AII-A2)	A	IGT39	A (AII-A2)	A (AII-A3)	A (AII-A2)	A
IGT9	A (AII-A2)	A (AII-A3)	A (AII-A2)	A	IGT40	A (AII-A2)	A (AII-A3)	A (AII-A2)	A
IGT10	B (BIII)	B (BIV-B3)	B	A+B	IGT41	A (AII-A2)	A (AII-A2)	A (AII-A2)	A
IGT11	A (AII-A2)	A (AII-A3)	A (AII-A2)	A+B	IGT7H	A (AII-A2)	A	A (AII-A2)	A
IGT12	A (AII-A2)	A (AII-A3)	A (AII-A2)	A+B	IGR12	–	B (BIV-B3)	B	B
IGT13	A (AII-A2)	A (AII-A3)	A (AII-A2)	A+B	IGT52	B (BIII)	B (BIV)	B (BIII)	B
IGT14	A (AII-A2)	A (AII-A3)	A (AII-A2)	A+B	IGR81	A (AII-A2)	A (AII-A3)	A (AII-A2)	A+B
IGT15	B (BIII/BIII-like)	B (BIV)	B	B	**IGT93**	**B (BIII)**	**A**	**–**	B
**IGT16**	**A (AII-A2)**	**B (BIV)**	**–**	B	IGR101	B (BIII)	B (BIV)	B	A+B
IGT17	B (BIII/BIII-like)	B (BIV-B3)	B	A+B	IGT110	B	B (BIV)	B (BIV-like)	A+B
IGT18	B (BIII/BIII-like)	B (BIV)	B	A+B	IGT117	–	A (AII-A3)	–	A+B
IGT19	A (AII-A2)	A (AII-A2)	A (AII-A2)	A+B	IGT143	A (AII-A2)	A (AII-A3)	A (AII-A2)	A+B
IGT20	A (AII-A2)	A (AII-A3)	A (AII-A2)	A	IGT152	–	B (BIV)	–	B
IGT21	A (AII-A2)	A (AII-A3)	A (AII-A2)	A	IGT164	B (BIII)	B (BIV-B3)	B (BIV-like)	B
IGT22	A (AII-A2)	A (AII-A3)	A (AII-A2)	A+B	**IGT165**	**A (AII-A2)**	**B (BIV)**	**–**	**–**
IGT23	B (BIII/BIII-like)	B (BIV-B3)	B (BIII-like)	B	IGT182	B (BIII)	B (BIV)	B (BIII)	B
IGT24	B (BIII)	B (BIV-B3)	B	B	IGR197	B	B (BIV)	B (BIII)	A+B
IGT25	A (AII-A2)	A (AII-A3)	A (AII-A2)	A+B	**IGT213**	–	**A (AII-A3)**	**B (BIV-like)**	B
IGT26	A (AII-A2)	A (AII-A3)	A (AII-A2)	A+B	IGR287	A (AII-A2)	A (AII-A3)	A (AII-A2)	A+B
IGT27	B (BIII)	B (BIV-B3)	B	B	IGA305	B (BIII)	B (BIV)	B (BIV)	A+B
IGT28	B (BIII)	B (BIV-B3)	B	B	IGA340	A (AII-A2)	A (AII-A3)	A (AII-A2)	A+B
IGT29	A (AII-A2)	A (AII-A2)	A (AII-A2)	A+B	**IGR386**	**–**	**B (BIV)**	**A (AII-A2)**	A+B
IGT30	A (AII-A2)	A (AII-A3)	A (AII-A2)	A+B	IGA458	–	B (BIV)	–	B
IGT31	A (AII-A2)	A (AII-A2)	A (AII-A2)	A+B	IGR519	B (BIII)	B (BIV)	B	B

Discordant assemblage isolates are in bold. The dash (–) indicates no amplification.

**Table 3:** Identification of assemblages A and B based on *tpi, bg*, and *gdh* loci, multilocus genotypes (MLGs) and mixed infection according to *tpi* A and B assemblage-specific primers.

**Genes**	**Assemblages** ***n*** **(%)**			**Total**
	**A**	**B**	**A + B**	
*tpi*	29 (53.7)	25 (46.3)	–	54 (87.1)
*bg*	28 (45.2)	34 (54.8)	–	62 (100)
*gdh*	28 (53.8)	24 (46.2)	–	52 (83.9)
MLGs	24 (52.2)	22 (47.8)	–	46 (74.2)
*tpi*-mixed	9 (14.5)	23 (37.1)	28 (45.2)	60 (96.8)

The amplification of the *bg* locus was successful in all 62 isolates (100%), whereas 54 (87.1%) and 52 (83.9%) of the isolates were successfully amplified by targeting *tpi*, and *gdh* loci, respectively. Consequently, 48 and 10 isolates were characterized in three and two loci, respectively, and four isolates were only amplified at the *bg* locus. The sequence analysis of the *tpi* and *bg* genes identified 29 (53.7%) and 28 (45.2%) isolates as assemblage A, and 25 (46.3%) and 34 (54.8%) isolates as assemblage B, respectively. Consistently, the amplification of the *gdh* gene identified 28 (53.8%) and 24 (46.2%) of *G. duodenalis* isolates as assemblages A and B, respectively (Table 3).

The MLG typing of *G. duodenalis* isolates from infected individuals without (*n* = 50) and with (*n* = 12) clinical symptoms showed 21 (42.0%; 95% CI 29.4–55.8) and 22 (44.0%; 95% CI 31.2–57.7), and 7 (14.0%; 95% CI 7.0–26.2) of the asymptomatic individual were infected with assemblages A, B, and discordant assemblages, respectively. Furthermore, symptomatic individuals were equally infected with assemblages A (5) and B (5) (41.7%; 95% CI 19.3–68.0), while discordant assemblages were detected in 2 (16.7%; 95% CI 4.7–44.8). There was no statistical association between assemblages and symptoms.

### Molecular characterization of *G. duodenalis* isolates within assemblage A: The sole occurrence of AII

**Figure 1:**
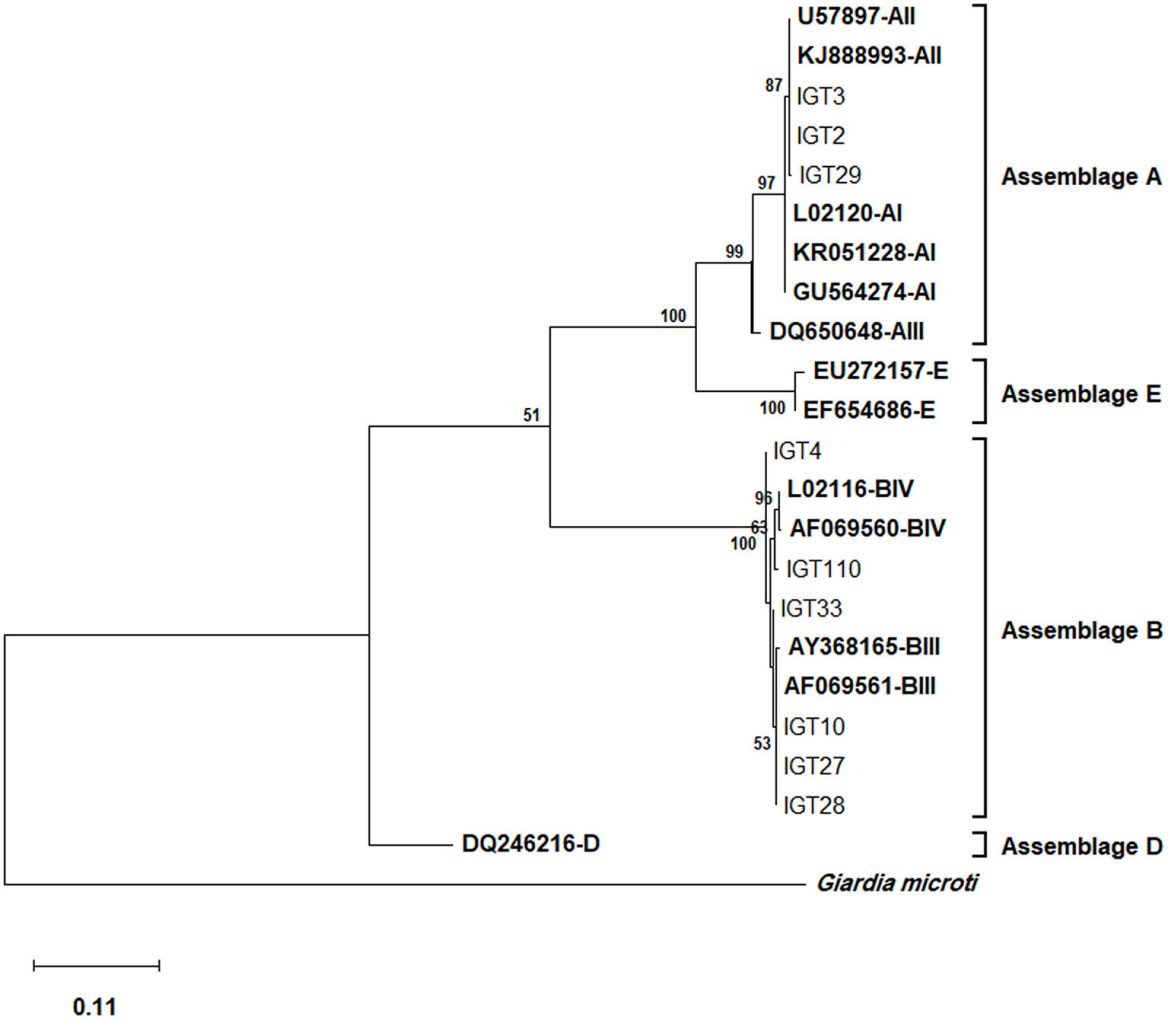
The phylogenetic analysis of the *tpi* gene nucleotide sequences of *Giardia duodenalis* using the Maximum Likelihood (ML) method and Tamura 3-parameter model (38) (T92 + G + I). The analysis involved 23 nucleotide sequences: Nine representative nucleotide sequences of *tpi* retrieved from this study (LC183913–LC183966) compared with 13 reference sequences of known assemblages from Genbank (indicated in bold) with *Giardia microti* as an outgroup. The percentage of trees in which the associated taxa clustered together (achieved from 1000 replicates) is shown next to the branches, only bootstrap values >50% are demonstrated. A discrete Gamma distribution was used to model evolutionary rate differences among sites [5 categories (+*G*, parameter = 9.6789)]. The rate variation model allowed for some sites to be evolutionarily invariable [(+*I*), 27.96% sites]. The scale bar represents substitutions per nucleotide. The final dataset contained 456 positions. Evolutionary analyses were conducted in MEGA X (39).

Multiple sequence alignments and the phylogenetic tree construction based on the *tpi* locus classified all 29 assemblage A isolates as sub-assemblage AII, subtype A2 (AII/A2), placing these isolates in a single cluster with AII/A2, as evidenced by a strong bootstrap value (Figure 1). Furthermore, the multiple alignments based on the *tpi* gene sequences also identified three-nucleotide substitution patterns, where 27 isolates, as represented by IGT2 (LC183914), had a 100% homology with the AII reference sequences (U57897, KJ888993). In comparison, two isolates demonstrated a single nucleotide substitution at positions 536 (T → G) and 445 (G → A) (Table 4).

**Table 4:** Triosephosphate isomerase (*tpi*) multiple alignment sequence isolates in this study with reference sequences retrieved from GenBank, representing position of intra-genotypic substitutions in assemblages and sub-assemblages of assemblages A and B.

	**Isolates/GenBank accession no**.	**Nucleotide position from the start of the gene**															
**Assemblage A**		**129**	**399**	**445**	**536**												
**AI**	**KR051228**	**T**	**C**	**G**	**G**												
**AI**	**L02120**	.	.	.	**T**												
**AII-A2**	**U57897**	**C**	**T**	.	**T**												
**AII-A2**	**KJ888993**	**C**	**T**	.	**T**												
**AII-A2**	IGT2, 5, 8, 9, 11–14, 16, 19–22, 25, 26, 30, 31, 37, 39–41, 7H, 143, 165; IGR81, 287; IGA340	C	T	.	T												
**AII**	IGT3	C	T	.	.												
**AII**	IGT29	C	T	A	T												
**Assemblage B**		**39**	**91**	**141**	**159**	**162**	**165**	**168**	**210**	**269**	**304**	**393**	**402**	**420**	**429**	**504**	**534**
**BIII**	**AY368165**	**G**	**C**	**C**	**G**	**G**	**C**	**C**	**G**	**G**	**G**	**T**	**A**	**T**	**G**	**C**	**C**
**BIII**	**AF069561**	**-**	.	.	.	.	.	.	.	.	**A**	**C**	.	.	.	.	**–**
**BIV**	**L02116**	**A**	**T**	.	.	.	**T**	**T**	**A**	.	**A**	**C**	.	.	.	.	**T**
**BIV**	**AF069560**	**A**	**T**	.	.	.	**T**	**T**	**A**	.	**A**	**C**	.	.	**A**	.	**–**
BIII/BIII-like	IGT1, 15, 17, 18	.	.	.	.	.	**Y**	.	.	.	A	C	.	.	.	.	.
B	IGT4	A	T	T	.	A	.	.	.	.	A	C	.	.	.	T	.
BIII	IGT7	.	.	.	.	.	.	.	.	.	**R**	**Y**	.	.	.	.	.
BIII	IGT10	.	.	.	.	.	.	.	.	.	A	C	.	.	.	.	T
BIII/BIII-like	IGT23	**R**	.	.	.	**R**	.	.	.	.	A	C	.	.	.	.	.
BIII	IGT24	**R**	.	.	**R**	.	.	.	.	.	A	C	.	.	.	.	.
BIII	IGT27	A	.	.	.	.	.	.	.	.	A	C	.	.	.	.	.
BIII	IGT28, 32, 35, 36, 52, 93, 164, 182; IGR101; IGA305	.	.	.	.	.	.	.	.	.	A	C	.	.	.	.	.
BIII-like	IGT33	.	.	.	.	A	.	.	.	.	A	C	.	.	.	.	.
BIII/BIII-like	IGT34	.	.	.	.	**R**	.	.	.	.	A	C	.	.	.	.	.
BIII	IGT35	.	.	.	.	.	.	.	.	.	A	C	.	.	.	.	.
B	IGT110	A	T	.	.	.	T	.	.	A	A	C	G	.	.	.	.
B	IGR197	**R**	.	.	.	.	T	**Y**	.	.	A	C	.	.	.	.	.
BIII	IGR519	.	.	.	.	.	.	.	.	.	A	C	.	**Y**	.	.	.

Accession numbers of the isolates used as sub-assemblage reference isolates are given in bold. Numbers in bold represent nucleotide substitutions from the start of the gene, which differentiate between sub-assemblages introduced by Weilinga and Thompson (15) position and breakdown of intra-genotypic substitutions. Heterogeneous positions are indicated by standard IUPAC codes in bold. Dots denote nucleotide homology with the AI (KR051228) or BIII (AY368165) reference sequences.

**Figure 2:**
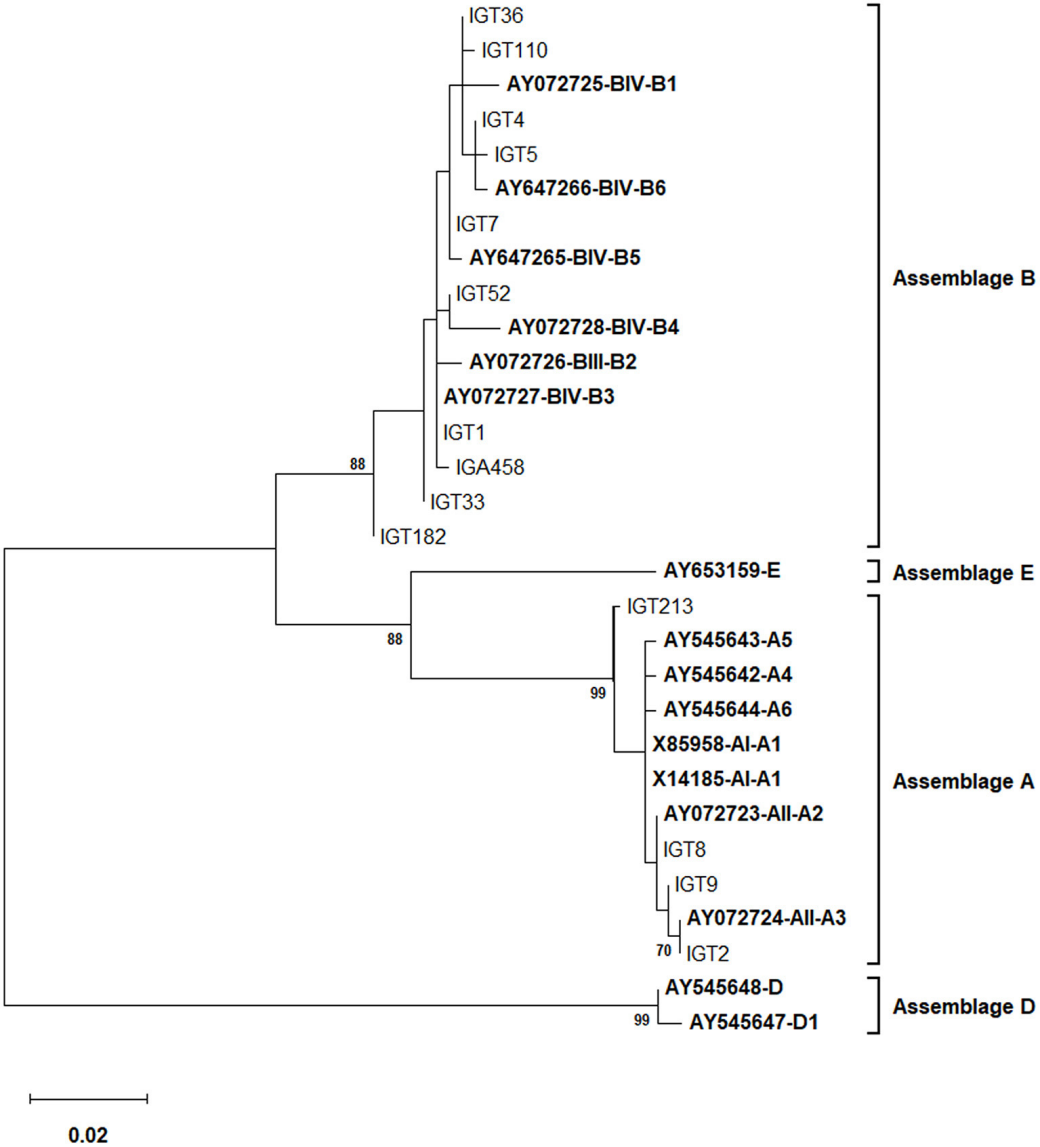
The phylogenetic analysis of the *bg* gene nucleotide sequences of *Giardia duodenalis* using the Maximum Likelihood method (ML) and Tamura 3-parameter model (38) (T92 + G + I). The analysis involved 30 nucleotide sequences: 14 representative nucleotide sequences of *bg* retrieved from this study (LC183967–LC184028) compared with 16 reference sequences of known assemblages from Genbank which are indicated in bold. The percentage of trees in which the associated taxa clustered together (achieved from 1,000 replicates) is shown next to the branches, only bootstraps values >50% are demonstrated. A discrete Gamma distribution was used to model evolutionary rate differences among sites [5 categories (+G, parameter = 0.1294)]. The rate variation model allowed for some sites to be evolutionarily invariable [(+I), 42.59% sites]. The scale bar represents substitutions per nucleotide. The final dataset included 506 positions. Evolutionary analyses were conducted in MEGA X (39).

As depicted in Figure 2, the phylogenetic analysis based on the *bg* locus placed five isolates (representative: IGT8) in a single cluster with sub-assemblage AII, subtype A2 (AII/A2), whereas 18 isolates (representative: IGT2) were classified in a clade together with sub-assemblage AII, subtype A3 (AII/A3). The *bg* locus sequence analysis found six distinct nucleotide substitution patterns, with two isolates displaying sequence homology to subtype A3 with a single substitution at the nucleotide position 460 (T → C) and one isolate with two nucleotide substitutions at positions 303 (A → G) and 460 (T → C). Furthermore, two *G. duodenalis* assemblage A isolates had multiple nucleotide substitutions and overlapping nucleotide peaks in fifteen positions, preventing them from being further characterized at the sub-assemblage/subtype level, as shown in Table 5.

**Table 5:** β -giardin (*bg*) multiple alignment sequence isolates in this study with reference sequences retrieved from GenBank, representing position of intra-genotypic substitutions in sub-assemblages of assemblages A and B.

**Isolates/GenBank accession no**.					**Nucleotide position from the start of the gene**																					
**Assemblage A**					**177**	**204**	**216**	**273**	**285**	**303**	**333**	**357**	**393**	**417**	**432**	**435**	**460**	**468**	**498**	**516**	**534**	**541**	**564**	**567**	**606**	**624**
**AII-A2**	**AY072723**				**C**	**A**	**G**	**G**	**C**	**A**	**T**	**G**	**T**	**T**	**G**	**T**	**C**	**T**	**A**	**C**	**G**	**G**	**C**	**G**	**T**	**G**
**AI-A1**	**X85958**				.	.	.	.	.	.	.	.	.	.	.	.	.	.	.	.	.	.	.	.	**C**	.
**AII-A3**	**AY072724**				.	.	.	.	.	.	.	.	.	.	.	.	**T**	**C**	.	.	.	.	.	.	.	.
**AII-A4**	**AY545642**				.	.	.	.	.	.	.	.	.	.	.	.	.	.	.	.	.	**A**	.	.	**C**	.
AII-A3	IGT2,11-14, 20-22, 25-26, 30, 37, 39-40, 117, 143; IGR81,287				.	.	.	.	.	.	.	.	.	.	.	.	T	C	.	.	.	.	.	.	.	.
AII**-**A2	IGT8, 19, 29, 31, 41				.	.	.	.	.	.	.	.	.	.	.	.	.	.	.	.	.	.	.	.	.	.
AII-A3	IGT9; IGA340				.	.	.	.	.	.	.	.	.	.	.	.	.	C	.	.	.	.	.	.	.	.
AII-A3	IGT213				.	.	.	.	.	G	.	.	.	.	.	.	.	C	.	.	.	.	.	.	.	.
A	IGT93				**T**	.	**R**	.	.	.	**Y**	.	.	**Y**	A	C	.	**Y**	.	.	**R**	.	.	**S**	.	S
A	IGT7H				.	**R**	.	**R**	**Y**	**R**	.	**Y**	**Y**	**Y**	A	.	T	C	**M**	**Y**	**R**	.	T	**S**	.	.
																									
**Assemblage B**		**210**	**228**	**273**	**303**	**327**	**354**	**357**	**384**	**393**	**432**	**435**	**438**	**450**	**471**	**538**	**541**	**550**	**564**	**594**	**609**	**610**	**636**	**639**	**645**	**648**
**BIII-B2**	**AY072726**	**C**	**A**	**G**	**G**	**C**	**C**	**C**	**C**	**C**	**A**	**C**	**C**	**C**	**C**	**G**	**G**	**G**	**T**	**G**	**C**	**G**	**G**	**G**	**C**	**G**
**BIV-B3**	**AY072727**	.	.	**A**	.	.	.	**T**	.	.	.	.	.	.	.	.	.	.	.	.	.	.	.	.	.	.
**BIV-B1**	**AY072725**	**T**	.	**A**	.	**T**	**T**	**T**	.	.	.	.	**T**	.	.	.	.	.	.	.	.	.	.	.	.	**A**
**BIV-B4**	**AY072728**	.	**G**	**A**	.	.	**T**	**T**	.	.	.	.	.	.	.	.	.	.	**C**	.	**T**	.	.	.	**T**	.
**BIV-B5**	**AY647265**	.	.	**A**	.	.	**T**	**T**	.	.	.	.	.	.	**T**	.	.	.	.	.	.	.	.	.	.	.
**BIV-B6**	**AY647266**	**T**	.	**A**	.	.	.	**T**	.	.	.	.	.	.	.	.	.	.	.	.	.	.	.	.	.	.
BIV-B3	IGT1,3,6,10,17,23,24,27,28,35,164; IGR12	.	.	A	.	.	.	T	.	.	.	.	.	.	.	.	.	.	.	.	.	.	.	.	.	.
BIV-B6	IGT4	T	.	A	.	.	.	T	.	.	.	.	.	.	.	.	.	.	.	.	.	.	.	.	.	.
BIV	IGT5	T	.	A	.	.	.	T	.	.	.	.	.	.	.	.	.	.	.	.	.	A	.	.	.	.
BIV	IGT7,15,16,34	.	.	A	.	.	T	T	.	.	.	.	.	.	.	.	.	.	.	.	.	.	.	.	.	.
BIV	IGT18; IGR197	.	.	**R**	.	.	.	T	.	.	.	.	.	.	.	.	.	.	.	.	.	.	.	.	.	.
BIV	IGT32	.	.	A	.	.	.	T	.	.	.	.	.	.	.	.	.	.	**R**	.	.	.	.	.	.	.
BIV	IGT33	.	.	A	.	.	.	T	.	T	.	.	.	.	.	.	.	.	.	.	.	.	.	.	.	.
BIV	IGT36, 38; IGR101,386	T	.	A	.	.	T	T	.	.	.	.	.	.	.	.	.	.	.	.	.	.	.	.	.	.
BIV	IGT52	.	G	A	.	.	.	T	.	.	.	.	.	.	.	.	.	.	.	.	.	.	.	.	.	.
BIV	IGT110; IGA305	T	.	A	.	.	T	T	.	.	.	.	.	.	.	.	.	.	.	A	.	.	.	.	.	.
BIV	IGT152	.	.	**R**	.	.	.	T	.	.	.	.	.	.	.	T	C	C	.	.	.	.	.	.	.	.
BIV	IGT165	**Y**	.	**R**	**R**	.	T	**K**	T	T	.	.	.	.	.	.	.	.	.	.	.	.	.	.	.	.
BIV	IGT182	.	.	A	.	.	T	T	T	T	G	T	.	.	.	.	.	.	.	.	.	.	.	.	.	.
BIV	IGA458	.	.	A	.	.	.	T	.	.	.	.	.	T	.	.	.	.	.	.	.	.	.	.	.	.
BIV	IGR519	.	.	**R**	.	.	.	T	.	.	.	.	.	.	.	.	.	.	.	.	.	.	A	T	.	.

Accession numbers of the isolates used as sub-assemblage reference isolates are included in bold. Numbers in bold represent nucleotide substitutions from the start of the gene, which differentiate between sub-assemblages introduced by Weilinga and Thompson and Cacciò et al. (15, 17) position and breakdown of intra-genotypic substitutions. Heterogeneous positions are indicated by standard IUPAC codes in bold. Dots indicate nucleotide identity to the AII (AY072723) or BIII (AY072726) reference sequences.

**Figure 3:**
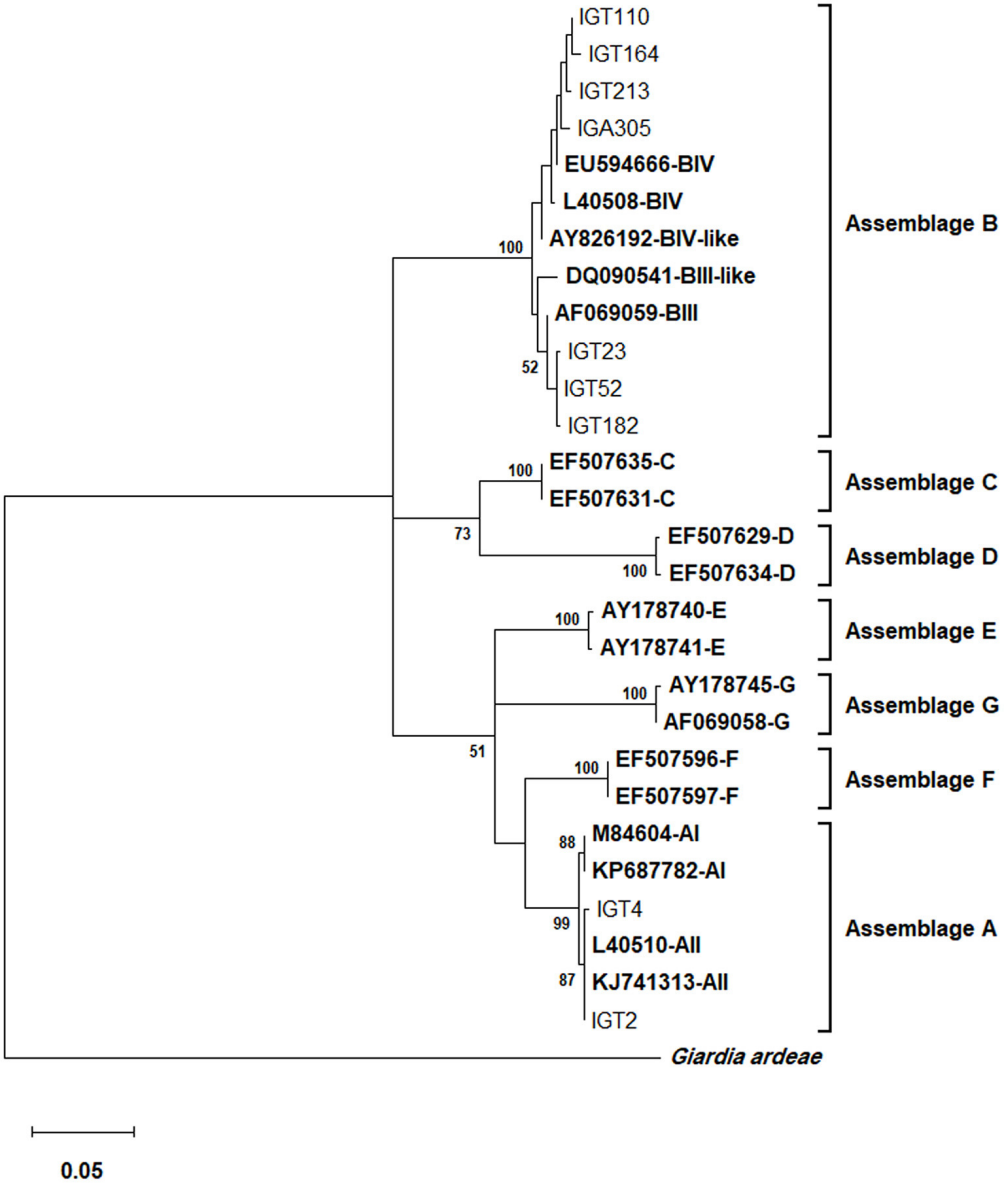
The phylogenetic analysis of the *gdh* gene nucleotide sequences of *Giardia duodenalis* using the Maximum Likelihood method and Tamura 3-parameter model (38) (T92 + G + I model). This analysis involved 29 nucleotide sequences: Nine representative nucleotide sequences of *gdh* retrieved from this study (LC184423–LC184474) compared with 19 reference sequences of known assemblages from Genbank (indicated in bold) with *Giardia ardeae* as an outgroup. The percentage of trees in which the associated taxa clustered together (achieved from 1,000 replicates) is shown next to the branches, only bootstraps values >50% are demonstrated. A discrete Gamma distribution was used to model evolutionary rate differences among sites [5 categories (+*G*, parameter = 0.4823)]. The rate variation model allowed for some sites to be evolutionarily invariable [(+*I*), 36.49% sites]. The scale bar represents substitutions per nucleotide. A total of 433 positions were evaluated in the final dataset. Evolutionary analyses were conducted in MEGA X (39).

Based on the *gdh* locus sequence analysis, the phylogenetic tree construction placed all 28 assemblage A isolates in a single cluster with a sub-assemblage/subtype AII/A2 (L40510), with a 99% bootstrap value (Figure 3). Furthermore, the multiple alignments using *gdh* sequences also demonstrated that 27 isolates possessed a 100% identity with the A2 subtype (L40510) of *G. duodenalis*, whereas an isolate (i.e., IGT4) showed a single substitution (A → G) at the nucleotide position 562 (Table 6).

**Table 6:** Glutamate dehydrogenase (*gdh*) multiple alignment sequence isolates in this study with reference sequences retrieved from GenBank, representing the position of intra-genotypic substitutions in sub-assemblages of assemblages A and B.

	**Isolates/GenBank accession no**.				**Nucleotide position from the start of the gene**																
**Assemblage A**		**562**	**603**	**621**
**AII-A2**	**L40510**	**A**	**C**	**T**	
**AI-A1**	**M84604**	.	**T**	**C**	
AII-A2	IGT2, 8-9, 11-14, 19-22, 25-26, 29-31, 37-41, 7H, 143; IGA340; IGR81, 287, 386	.	.	.	
AII-A2	IGT4	**G**	.	.	
																				
**Assemblage B**		**279**	**297**	**309**	**357**	**359**	**360**	**375**	**405**	**429**	**432**	**447**	**465**	**519**	**540**	**546**	**561**	**597**	**612**	**636**	**666**
**BIII**	**AF069059**	**C**	**C**	**C**	**T**	**C**	**G**	**G**	**G**	**T**	**C**	**T**	**C**	**C**	**C**	**C**	**C**	**C**	**G**	**T**	**T**
**BIII-like**	**DQ090541**	.	**T**	.	**C**	.	.	.	.	.	.	**C**	.	**T**	.	.	.	**T**	**A**	.	**-**
**BIV**	**L40508**	.	.	**T**	.	.	.	.	.	**C**	.	**C**	.	.	**T**	.	**T**	.	**A**	.	.
**BIV**	**EU594666**	.	.	**T**	**C**	.	.	.	.	**C**	.	**C**	.	.	**T**	.	**T**	.	**A**	.	**C**
**BIV-like**	**AY826192**	.	.	**T**	**C**	.	.	.	.	**C**	.	**C**	.	.	**T**	.	.	.	.	.	.
B	IGT1, IGT17	.	.	**Y**	.	.	.	.	.	**Y**	.	**Y**	.	.	.	**Y**	.	.	.	.	.
B	IGT7	.	.	**Y**	.	.	.	.	.	**Y**	.	.	.	.	.	.	.	.	.	.	.
B	IGT10	.	.	**Y**	.	.	.	.	.	**Y**	.	**Y**	.	**Y**	**Y**	.	.	.	**R**	.	.
B	IGT15	.	.	**W**	C	.	.	**R**	**R**	C	.	C	.	.	.	.	.	.	A	.	.
B	IGT18	.	.	**W**	**Y**	.	.	.	.	**Y**	.	**Y**	.	.	**Y**	.	.	.	A	.	C
BIII-like	IGT23	.	.	T	.	.	.	.	.	.	.	.	.	.	.	.	.	.	.	.	C
BIII	IGT24	.	.	**Y**	.	.	.	.	.	.	.	.	.	.	.	.	.	.	.	.	C
BIII	IGT27	.	.	**Y**	.	.	.	.	.	.	.	.	.	.	.	.	.	.	.	.	.
B	IGT28	.	.	**Y**	.	.	.	.	.	.	.	.	.	.	.	.	.	.	**R**	.	C
B	IGT32	.	.	.	**Y**	.	.	.	.	C	**Y**	.	.	**Y**	.	.	.	.	A	.	C
B	IGT34	.	.	T	C	.	.	.	.	**Y**	.	**Y**	**Y**	.	.	.	.	**Y**	A	.	C
B	IGT35	.	.	**Y**	**Y**	.	.	.	.	**Y**	.	**Y**	.	.	.	.	.	.	A	.	C
B	IGT36	.	.	T	C	.	A	.	.	**Y**	.	**Y**	.	.	.	.	.	.	A	.	C
B	IGR12	.	.	.	.	.	.	.	.	.	.	.	.	.	.	.	.	.	**R**	.	C
BIII	IGT52	.	.	.	.	.	.	.	.	.	.	.	.	.	.	.	.	.	.	.	C
B	IGR101	.	.	**Y**	.	.	.	.	.	.	.	**Y**	.	**Y**	**Y**	.	.	.	**R**	.	C
BIV-like	IGT110	.	.	T	C	.	.	.	.	C	.	C	.	.	T	.	.	.	A	C	C
BIV-like	IGT164	.	.	T	.	.	.	.	.	C	.	C	.	.	T	.	.	.	.	C	C
BIII	IGT182	.	.	.	.	.	.	.	.	.	.	.	.	T	.	.	.	.	.	.	C
BIII	IGR197	.	.	.	.	.	.	.	.	.	.	.	.	**Y**	.	**Y**	.	.	.	.	C
BIV-like	IGT213	.	.	T	C	T	.	.	.	C	.	C	.	.	T	.	.		A	.	C
BIV	IGA305	T	.	T	C	.	.	.	.	C	.	C	.	.	T	.	T	T	A	.	C
B	IGR519	.	.	.	**Y**	.	.	.	.	.	.	.	.	.	.	.	.	.	**R**	.	C

Accession numbers of the isolates used as sub-assemblage reference isolates are included in bold. Numbers in bold represent nucleotide substitutions from the start of the gene, which differentiate between sub-assemblages introduced by Weilinga and Thompson (15) position and breakdown of intra-genotypic substitutions. Heterogeneous positions are indicated by standard IUPAC codes in bold. Dots indicate nucleotide identity to the AII (L40510) or BIII (AF069059) reference sequences.

### Molecular characterization of *G. duodenalis* isolates within assemblage B

Multiple sequence alignments based on the *tpi* locus amplification identified 25 isolates as assemblage B of *G. duodenalis*, representing 14 distinct nucleotide substitution patterns (Table 4). Fifteen isolates (15/25) were characterized as sub-assemblage BIII, of which 10 isolates had a 100% identity with the reference sequence AF069561. Moreover, two isolates showed a single nucleotide substitution at positions 534 (IGT10) and 39 (IGT27), which were not in the sub-assemblage-defining positions (hotspot sites) (15). Sequences of 10 isolates (10/25, 40%) presented overlapping nucleotide peaks in eight positions. Only two were in hotspot sites, so double peaks did not interfere with the characterization of sub-assemblage BIII in three isolates. The comparative sequence analysis between 14 distinct nucleotide substitution patterns and the reference sequences representing BIII and BIV categorized one isolate as BIII-like, and six isolates showed overlapping nucleotide peaks at one or two positions could classify the isolates as BIII/BIII-like of *G. duodenalis*. Further characterization of the remaining three isolates initially identified as assemblage B was not attainable at sub-assemblage levels (Table 4). As shown in Figure 1, the phylogenetic analysis showed the monophyletic group of assemblage B with bootstrap support of 100%.

Multiple sequence alignments based on the *bg* locus confirmed that all 34 isolates initially identified as assemblage B belonged to the BIV sub-assemblage of *G. duodenalis*, representing a total of 15 nucleotide sequences patterns (Table 5). In addition, two nucleotide substitution patterns representing 12 isolates (i.e., IGT1) and one isolate (IGT4) showed a 100% identity with the B3 (AY072727) and B6 (AY647266) subtype reference sequences of *G. duodenalis*, respectively. Sequences of six isolates (6/34, 17.6%) presenting overlapping nucleotide peaks in five positions were not in hotspot sites, so double peaks did not interfere characterization of sub-assemblage BIV. The nucleotide heterogeneity and genotype characterization of all 34 isolates are detailed in Table 5. The phylogenetic tree construction based on the *bg* locus clustered all assemblage B isolates of *G. duodenalis* in a single clade, with bootstrap support of 88% (Figure 2).

Further sequence alignment based on the *gdh* locus amplification identified 23 nucleotide substitution patterns, representing 24 assemblage B *G. duodenlais* isolates (Table 6). Nucleotide sequences of 17 isolates (17/24, 70.8%) presenting double nucleotide peaks in 13 positions mostly were in hotspot sites. Ten isolates were classified at sub-assemblage levels, whereas fourteen isolates exhibited nucleotide substitutions or ambiguous nucleotides at sub-assemblage–defining positions and could not be further characterized at sub-assemblage levels (Table 6). The phylogenetic analysis confirmed that the assemblage B clinical isolates all clustered in a monophyletic clade, supported by a 100% bootstrap value, as shown in Figure 3.

Finally, the sub-assemblage classification of assemblage B isolates by the three markers revealed inconsistent genotyping results at the intra-assemblage level: assemblage B isolates were tentatively classified as sub-assemblages BIII (*tpi* locus), BIV (*bg* locus), and BIII or BIV (*gdh* locus) (Table 2).

### Multilocus genotyping of *G. duodenalis* isolates

**Figure 4:**
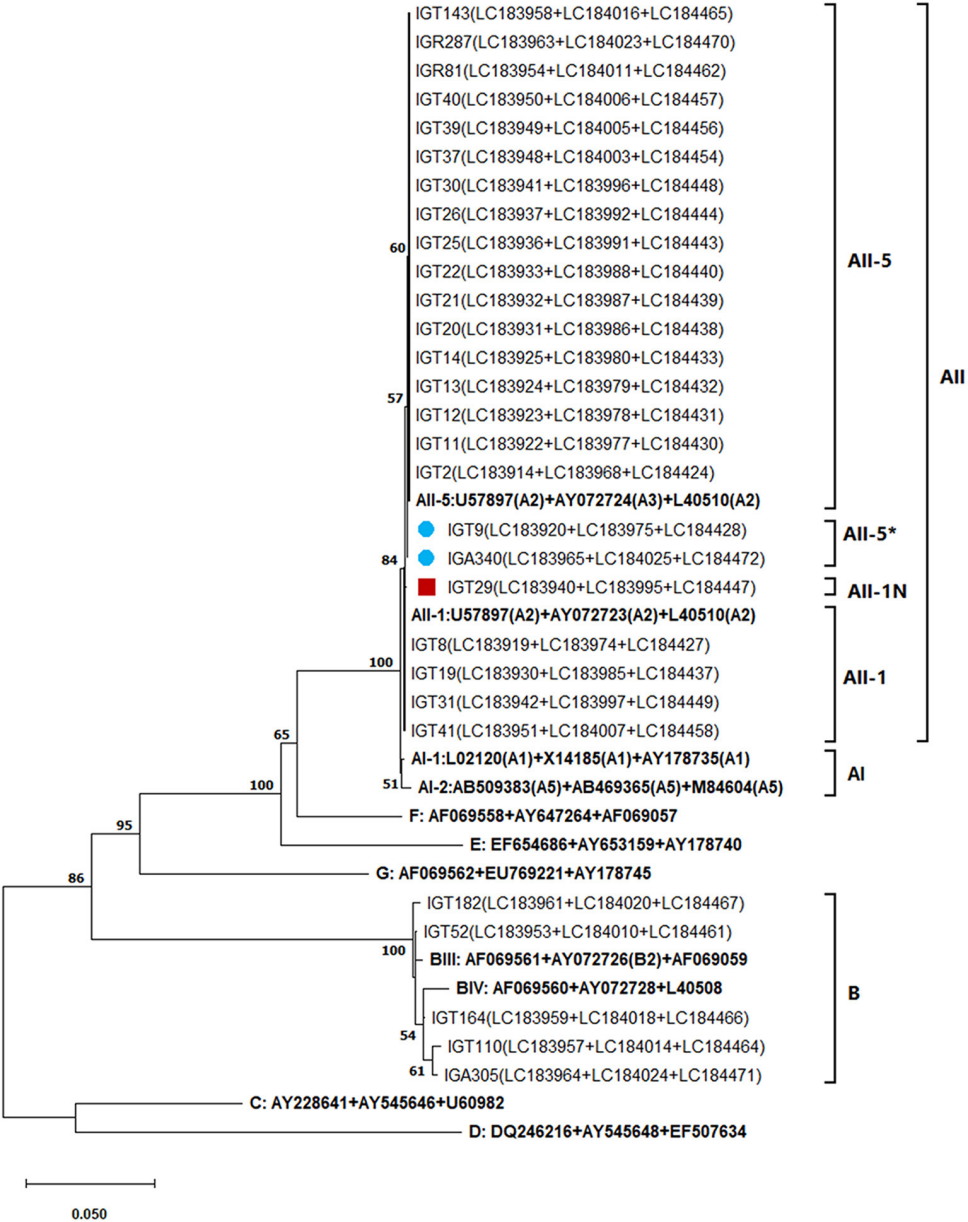
The phylogenetic analysis of the concatenated *tpi, bg*, and *gdh* nucleotide sequences of *Giardia duodenalis* using the maximum likelihood method (ML) and Tamura 3-parameter model (38) (T92 + G + I). The analysis involved 40 nucleotide sequences: 29 concatenated (*tpi* + *bg* + *gdh*) nucleotide sequences retrieved from this study compared with 11 known multilocus genotype reference sequences reported in previous studies (17, 40, 41) are indicated in bold. The red-filled square represents the new MLG of assemblage A reported in this study and the blue-filled circles indicate the one nucleotide substitution in A3 compared with the AII-5 MLG of assemblage A based on the modified numerical MLG reviewed in Cai et al. (4). The final dataset contained 1,395 positions. The percentage of trees in which the associated taxa clustered together (achieved from 1,000 replicates) is shown next to the branches, only bootstraps values >50% are demonstrated. A discrete Gamma distribution was used to model evolutionary rate differences among sites [5 categories (+*G*, parameter = 0.3229)]. The rate variation model allowed for some sites to be evolutionarily invariable [(+*I*), 37.78% sites]. The scale bar represents substitutions per nucleotide. Evolutionary analyses were conducted in MEGA X (39).

Employing an MLST approach, forty-six *G. duodenalis* isolates were successfully amplified, sequenced, and genotyped using the *tpi, bg*, and *gdh* loci. The simultaneous sequence analysis of the three loci (*tpi* + *bg* + *gdh*) were combined for each isolate to obtain the corresponding concatenated sequences (37). Further sequence alignment and phylogenetic analysis on the 29 concatenated sequences with unambiguous (no double peak) positions revealed that 24 *G. duodenalis* isolates were assemblage A in four distinct haplotypes. Besides, five isolates were assemblage B with five haplotypes (Table 7; Figure 4). The phylogenetic tree construction using obtained concatenated sequences showed that those clinical isolates initially identified as assemblage A or assemblage B of *G. duodenalis* clustered in two monophyletic branches with robust bootstrap support of 100%, being completely separated from each other and the host-specific assemblages C to G (Figure 4). Moreover, 24 *G. duodenalis* isolates initially identified as assemblage A were further classified into four MLGs (Table 7; Figure 4). As a result, we identified four isolates as MLG AII-1, profile A2/A2/A2, and 17 isolates as MLG AII-5, profile A2/A3/A2. Furthermore, two isolates were referred to as MLG AII-5, which showed one nucleotide substitution compared with AY072724 (4, 40). These two MLGs have been previously reported (3, 4, 17). Interestingly, we identified one novel MLG for assemblage A isolates, whose MLG could not be classified based on previously-proposed nomenclature (4, 17, 41) (Table 7). More specifically, a single isolate (IGT29) was designated as MLG AII-1N, (Table 7). No further definite classification of assemblage B isolates based on identified MLGs was not possible, since additional information on the nomenclature of assemblage B MLGs is not currently available (Figure 4).

**Table 7:** Multilocus genotyping (MLG) and subtypes in *Giardia duodenalis* assemblage A–positive of Iranian isolates according to sequencing data from *tpi* (triosephosphate isomerase), *bg* (β -giardin), and *gdh* (glutamate dehydrogenase) loci.

**MLG**	**Subtype**			**No. of isolates** **(isolate code)**	**GenBank accession no**		
	***tpi***	***bg***	***gdh***		***tpi***	***bg***	***gdh***
AII-1	A2	A2	A2	**4** (IGT8, 19, 31, 41)	**U57897**, LC183919, LC183930, LC183942, LC183951	**AY072723**, LC183974, LC183985, LC183997, LC184007	**L40510**, **AY178737**, LC184427, LC184437, LC184449, LC184458
AII-1N	**A2** ^**n**^	A2	A2	**1** (IGT29)	LC183940	**AY072723**, LC183995	**L40510**, **AY178737**, LC184447
AII-5	A2	A3	A2	**17** (IGT2, 11–14, 20–22, 25-26, 30, 37, 39–40, 143; IGR81, 287)	**U57897**, LC183914, LC183922–5, LC183931–3, LC183936–7, LC183941, LC183948, LC183949–50, LC183958, LC183954, LC183963	**AY072724**, LC183968, LC183977–80, LC183986–8, LC183991–2, LC183996, LC184003, LC184005–6, LC184016, LC184011, LC184023	**L40510**, **AY178737**, LC184424, LC184430–3, LC184438–40, LC184443–4, LC184448, LC184454, LC184456–7, LC184465, LC184462, LC184470
AII-5*	A2	A3*	A2	**2** (IGT9, IGA340)	**U57897**, LC183920, LC183965	**KC313948**, LC183975, LC184025	**L40510**, **AY178737**, LC184428, LC184472

Superscript **n** indicates a novel nucleotide sequence in A2. *Indicates the nucleotide substitution in the A3 sequence.

## Discussion

The multilocus sequence typing (MLST) of cyst-positive *Giardia* isolates from infected individuals in Tehran was performed to classify assemblage and inter-and intra-assemblage genetic diversity of human *G. duodenalis* in Tehran, Iran, based on *tpi, bg*, and *gdh* genes. We identified one new MLG of assemblage A.

To the best of our knowledge, this is the first study conducted on *G. duodenalis*-infected individuals in Tehran, Iran, using MLST. However, the overwhelming majority of studies in Iran have reported the molecular characterization of *G. duodenalis* isolates based on the analysis of one locus (21–26, 42, 43) or two loci of *gdh* and *tpi* (44–46) or *gdh* and *bg* (47). The MLG data was reported for two *Giardia* isolates in the only multilocus analysis in southwestern Iran (48).

Multilocus sequencing results of *tpi, bg*, and *gdh* genes showed *G. duodenalis*-infected individuals in Tehran to be infected with assemblages A and B, agreeing with reports of human infection worldwide [reviewed in Feng and Xiao (3)]. Furthermore, assemblages A and B occurred at equal rates, similar to a *Giardia* MLG study performed in Malaysia (36). Although Ryan and Cacciò (7) reported that assemblage B is more prevalent than A in humans worldwide, the predominance of assemblage A was reported in previous studies in Iran (21, 22, 25, 47), Turkey (49), Iraq (50), Syria (51), Saudi-Arabia (52), Egypt (53), Thailand (54), Italy (34), the Czech Republic (55), and Ethiopia (56). The disparities might reflect geographical distribution, study populations and differing molecular tools and genes used, as the effect of loci was evident in the results of assemblage B in *bg* (~55%, 34/62) compared with the *tpi* (~46%, 25/54) and *gdh* (~46%, 24/52) genes in our study (Table 3).

Moreover, the amplification rate of these genetic loci differs, as about 60% of *bg* and *tpi* genes and 40–60% of *gdh* genes can be detected by most primers (3), possibly explaining the different rates of amplification of *bg* (100%), *tpi* (87.1%), and *gdh* (83.9%) observed in this study. These findings are in agreement with previous reports (3, 36). Although the majority of our isolates (77.4%) were consistent assemblage classification in three loci, 14.5% showed discordant assemblage typing, which was in agreement with previous studies on human and animal isolates (3, 36, 57, 58). As Cacciò and Ryan (16) suggested, this phenomenon might result from mixed infections in fecal samples or gene exchange between assemblages, also known as allelic sequence heterozygosity (ASH). A high level of ASH is reported in *Giardia* parasites, which have a tetraploid genome resulting from being a binucleated organism. Furthermore, the different levels of ASH have evaluated among *G. duodenalis* assemblages in which the ASH degree in assemblage B is about 10-fold higher than assemblage A isolates. On the other hand, it is usually challenging to distinguish between a high level of ASH or mixed infection when the DNA of cysts retrieved from fecal samples is typing (37).

Mixed assemblage infections have been commonly reported in humans and animals (17, 32, 58), and assemblage-specific PCR assays provide evidence that the prevalence of mixed assemblage infections is high in clinical specimens (32, 36). In this study, *tpi* assemblage-specific PCR assays showed a high prevalence (45.2%) of mixed infection of A and B assemblage in Tehran isolates, but less than reported in Malaysia (64%) (36), although greater than in Belgium (32.4%) (32) using the same primers. Co-infection by assemblage A and B has been previously reported in Iran based on PCR-RFLP of the *gdh* locus (21, 25, 43, 45).

Consistent with previously global reports (2, 4), we found that genotyping of assemblage A revealed low genetic polymorphism. Furthermore, AII was the only sub-assemblage identified with the greatest variation at the *bg* locus, followed by *tpi* and *gdh*, which could be reflecting the presence of double peak nucleotide positions in two assemblage A isolates at the *bg* locus. This finding contrasts with previous studies reporting the greatest variability in the *tpi* gene and lowest in the *bg* gene (15, 17). However, it agrees with Wegayehu et al. (59), who observed variation at the *bg* locus. AII, with the predominant subtype A2, is considered the most prevalent sub-assemblage in humans, whereas AI and AIII sub-assemblages are rarely reported [reviewed in (2, 3, 7)]. Identifying sub-assemblage AII, subtype A2 in the examined assemblage A isolates at the three loci, which is rarely found in other animals, suggests the potential of human-to-human transmission in the population studied. These results support previous findings of AII in the *gdh* locus that indicate potential anthroponotic transmission of *Giardia* in Tehran (21), Shiraz (22, 43), and Kashan (25). However, validating this hypothesis requires extensive molecular studies of *Giardia* isolates in animals and the environment, as well as considering the infection risk factors.

Higher genetic polymorphism of *G. duodenalis* in assemblage B than in A observed at the *bg, tpi*, and *gdh* loci in this study are consistent with previous reports (37, 41, 59–61). Furthermore, the higher genetic heterogeneity in those isolates representing the assemblage B of *G. duodenalis* was predominantly noticeable at the *gdh* locus (70.8%), followed by the *tpi* (40.0%) and *bg* (17.4%) loci which are in agreement with prior studies (17, 59). However, the finding contrasts with earlier observations (15), showing *tpi* to be the most polymorphic locus, with fewer polymorphisms observed in *bg* and *gdh* loci. Geurden et al. (32) reported high diversity at the *bg* locus and less at *gdh* and lowest at *tpi*, while Lecová et al. (55) reported the highest *gdh* followed by *bg* and *tpi*. These seemingly contradictory results can be potentially further explained by the differential selective pressures that ultimately determine the extent to which a given gene exhibits the genetic polymorphism. In contrast to assemblage A, subgrouping of assemblage B is not supported by phylogenetic analyses of nucleotide sequences of current genotyping loci (2, 3, 14). Sub-assemblage determination was not possible among all assemblage B isolates due to high nucleotide polymorphism with the heterogeneous nucleotide in the sequence, as has been reported (14, 41, 60). Inconsistency among the three markers in sub-assemblage B isolates was observed in this study as well as in earlier reports (32, 41, 55, 59, 62). This finding may reflect differences among the loci (59) or mixed infections with different sub-assemblages (37) or ASH (4, 7, 37).

To resolve the discrepancies among genetic markers, MLST of *Giardia*-positive samples was performed by combining the sequencing data of *tpi, bg*, and *gdh* loci, according to Cacciò et al. (17). As a result, the concatenated sequences of 24 A assemblages were classified into three MLGs: two (AII-1 and AII-5) MLGs frequently reported throughout the world (4, 17, 41, 55) and one novel (AII-1N) MLG, with one SNP in subtype A2 *tpi* locus, considering a single nucleotide mutation sufficient to designate a new subtype (16, 41, 63, 64). Therefore, since 2008, when Cacciò et al. (17) proposed a genotyping nomenclature system based on MLG analysis of the *tpi, bg*, and *gdh* loci, new MLGs have frequently been identified based on different combinations of *tpi, bg*, and *gdh* (4, 41, 55, 64). However, the presence of highly overlapping nucleotide peaks in the sequencing profiles and the broad genetic variability among and within the three target genetic loci made classifying assemblage B isolates in nominated MLGs impossible. It has been proposed that the high sequence variability and double peaks are due to the high degrees of ASH, genetic recombination through cryptic sex involving two nuclei of *Giardia*, true mixed infections, or a combination of those factors (4, 37, 59, 65). Therefore, as mentioned (3, 7, 37), MLST is useful for the typing of assemblage A of *G. duodenalis*, although MLGs grouping of assemblage B is more complex as a result of its high inter-and intra-sequence variability. In addition, as shown in the concatenated phylogenetic tree (Figure 4), the MLST is a practical tool for separating A and B isolates from each other and host-specific assemblages (C–G) and constructs host-specific clusters with high bootstrap support. Therefore, applying MLST allowed us to characterize *G. duodenalis* isolates circulating in Tehran and identify their genetic diversity.

The AII sub-assemblage is mainly reported in humans, considering that human and non-human primates are the predominant hosts of assemblage B and is much less frequent in wildlife and dogs (4, 7). Our identification of anthroponotic assemblages and sub-assemblages (B and AII) of *G. duodenalis* suggest that humans are likely a potential source of infection and person-to-person transmission probably occurs in Tehran. However, the main limitation of this hypothesis is the limited data on non-human giardiasis in Iran. To address this issue, comprehensive molecular studies to determine the genotype/subtype of *Giardia* infection of humans and companion and livestock animals that cohabit or occur in the same location, as well as environmental *G. duodenalis* isolates, are essential. Moreover, typing of assemblage B isolates should be performed separately using the MLST scheme with the newly identified best-performing genes developed in recent years to shed light on the transmission cycle of this mysterious parasite.

## Conclusions

Assemblages A and B are equally represented in *G. duodenalis*-infected individuals in the current study. The multilocus sequence analysis reveals genetic diversity in both assemblages and novel MLG of assemblage A. However, the lack of a solid consensus around the nomenclature of genetic variants within the assemblage B of *G. duodenalis* at the sub-assemblage levels is challenging. The multilocus sequencing is useful for typing assemblage A and discriminating assemblages of *G. duodenalis*. Applying MLST also provides insight into the genetic diversity of *G. duodenalis* isolates. Our findings suggest that *G. duodenalis* is potentially transmitted *via* a person-to-person route in Tehran, although further MLST of *Giardia* isolates from humans, companion animals, livestock, and the environment is recommended to elucidate the mode of transmission.

## Data availability statement

The datasets presented in this study can be found in online repositories. The names of the repository/repositories and accession number(s) can be found in the article/supplementary material.

## Ethics statement

The studies involving human participants were reviewed and approved by the Ethics Committee of Iran University of Medical Sciences (IUMS) with the code number: IR.IUMS.REC.1394.25787. Written informed consent to participate in this study was provided by the participants' legal guardian/next of kin.

## Author contributions

SH-H: methodology, validation, formal analysis, investigation, resources, data curation, visualization, and writing—original draft. ARM: methodology, validation, resources, and writing—review and editing. MM and NH: resources. SS-M: methodology, validation, formal analysis, and writing—review and editing. ER: conceptualization, methodology, validation, formal analysis, resources, data curation, writing—original draft, writing—review and editing, visualization, supervision, project administration, and funding acquisition. All authors contributed to the article and approved the submitted version.

## Funding

This study was financially supported by the Iran University of Medical Sciences under grant number 94-01-30-25787 to ER. Research in the Laboratory of Mucosal Immunology was supported by a startup fund (20344-8015) from the Department of Biomedical Sciences, School of Medicine and Health Sciences, the University of North Dakota to SS-M, a Dean's Meritorious Pilot Grant, School of Medicine and Health Sciences, the University of North Dakota to SS-M, and by NIH/NIGMSP20GM113123 to SS-M.

## Conflict of interest

The authors declare that the research was conducted in the absence of any commercial or financial relationships that could be construed as a potential conflict of interest.

## Publisher's note

All claims expressed in this article are solely those of the authors and do not necessarily represent those of their affiliated organizations, or those of the publisher, the editors and the reviewers. Any product that may be evaluated in this article, or claim that may be made by its manufacturer, is not guaranteed or endorsed by the publisher.
